# A novel KIAA0196 mutation in a Chinese patient with spastic paraplegia 8: A case report

**DOI:** 10.1097/MD.0000000000010760

**Published:** 2018-05-18

**Authors:** Limin Ma, Yingying Shi, Zhongcan Chen, Shujian Li, Weiwei Qin, Jiewen Zhang

**Affiliations:** a Department of Neurology, People's Hospital of Zhengzhou University; b Department of Neurology, Henan Provincial People's Hospital, Zhengzhou, China.

**Keywords:** gene mutation, HSP, KIAA0196, SPG8

## Abstract

**Rationale:**

We report a case of Spastic paraplegia 8 (SPG8) with a novel mutation of KIAA0196 gene.

**Patients concerns:**

A 12-year-old boy presented as ankle sprained, lower limb stiffness, abnormal gait since he was 5 years old.

**Diagnoses:**

The next generation sequence showed a novel c.1128delG (p.L376fs) mutation in KIAA0196 gene, the electromyography showed the pyramidal tract conduction dysfunction and deep sensory conduction abnormalities of lower limbs without motor neuron damage. The diagnose was SPG8.

**Interventions:**

Patient was gaven Baclofen treatment (30 mg/day, orally).

**Outcomes:**

At one year follow up, his symptoms didn’t improved.

**Lessons:**

We describe a novel KIAA0196 c.1128del.G (p.L376fs) mutation in a Chinese patient with SPG8. To our knowledge, it's the first frame delete mutation causing shift mutation of KIAA0196 gene, resulting in the earliest onset of SPG8 in the world. Gene sequencing is a powerful diagnostic tool to identify a causal mutation in genetically heterogeneous HSP.

## 1 Introduction

Spastic paraplegia (SPG), also known as hereditary spastic paraplegia (HSP), refers to a genetically and clinically heterogeneous group of neurodegenerative disorders featured with upper motor neuron atrophy.^[1]^ HSPs are categorized by a genetic classification scheme.^[2]^ SPG8 is caused by mutations in the *KIAA0196* gene on chromosome 8, which encodes the protein strumpellin.^[1]^ Although 77 different loci and 60 genes have been shown to be associated with HSPs,^[3]^ only 10 missense mutations and a gross deletion mutation have been identified as a cause of SPG8.^[1]^ The present study reported a novel KIAA0196 c.1128delG (p.L376fs) mutation in a Chinese patient with SPG8, which was also identified as the first frameshift mutation of *KIAA0196* gene associated with SPG8 in the world.

### 1.1 Consent statement

Informed consent was obtained from the patient for the publication of this study.

## 2 Case report

**Figure 1:**
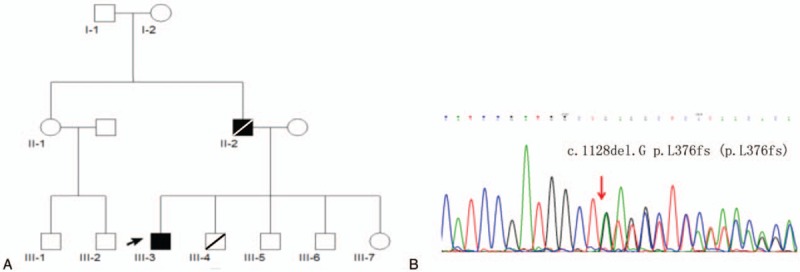
(A) Family tree of the KIAA0196 C.1128del.G p.L376fs mutation pedigree. The arrow indicates the proband (III-2), the square indicates male, the circle indicates female, the black symbol indicates affected family member, and the slashed symbol indicates the dead family member. (B) DNA sequencing chromatograph of exon 9 of the *KIAA0196* gene.

The proband (III-3) (Fig. 1A), a 12-year-old boy, visited Henan Provincial People's Hospital on February 23, 2016, with the chief complaint of “running difficulty for about 8 years and walking difficulty for about 1 year.” About 8 years ago, when he was 5 years old, his parents noticed that he sprained his ankle easily while running. After 3 years, stiffness in the lower limb with progressive slow running and abnormal gait was observed. Half a year ago, he developed occasional bladder-control problem. All the symptoms deteriorated gradually with time. The physical examination showed increased lower limb muscle tension, typical spastic gait, brisk reflexes, ankle clonus, decreased vibration sensation, and lower limb muscle atrophy, although his intelligence remained normal. The electromyography showed conduction dysfunction of the pyramidal tract and deep sensory conduction abnormalities of lower limbs without motor neuron damage. No abnormality in the laboratory examination was noted, including blood cell count, thyroid hormone level, erythrocyte sedimentation rate, C-reactive protein, folic acid, and vitamin B12 level. In summary, the diagnosis of the proband was determined as a pure HSP.

The proband's father (II-2) had a similar symptom presented with spastic gait in his 20s and died in his 30s because of accident. The proband's brother (III-4) died when he was 15 days old because of feeding difficulty. Other family members were normal.

As his father had similar symptoms, it was inferred that the disease was probably dominantly inherited. The next-generation sequence detection of HSP-related 59 genes from the proband was carried out. The c.1128del.G on exon 9 causing p.L376fs was found in the *KIAA0196* gene, which was validated by a Sanger sequencing (Fig. 1B). It was predicted that this variant was likely pathogenic, which could explain the symptoms of the patient. Consequently, the diagnosis of the proband was found to be SPG8 type. Baclofen treatment (30 mg/d, orally) was given to the proband for 1 year; however, his symptoms did not improve.

## 3 Discussion

HSP can be classified into pure and complicated forms.^[4]^ The pure HSPs are characterized by spasticity and motor deficit of the legs, corticospinal tract signs, which are often accompanied by hypertonic urinary disturbances and deep sensory impairment. Complex HSP forms are characterized by the presence of additional neurologic impairments,^[3]^ such as cognitive impairment, optic atrophy, cerebellar ataxia, peripheral nerve involvement, seizures, and extrapyramidal disturbances.^[4]^ Most of the autosomal dominant–HSPs are reported to present clinically as pure HSPs, whereas most of the AR-HSPs are complicated forms.^[5]^ SPG8 is an autosomal dominant form. According to the reported SPG8, except p.I226T that manifested as complex form, p.N471D and g.ex11–15del performed as unknown phenotype, and all the other 8 missense mutations acted as pure forms.^[1,6]^ The proband also acted as a pure form, which was in line with the previous studies.

HSP-type SPG8 is associated with relatively little interfamilial variability, but rather severe phenotype, and the age of disease onset for patients is usually 20 or 30 years.^[4]^ According to the reported SPG8 variant patients, the age of disease onset ranged from 10 to 60 years, mostly at 20 years, which was consistent with the previous studies. However, in this study, the age of disease onset of the proband was 5 years, which is younger than ever reported. This may be because the frameshift gives rise to a more serious change in protein structure and sequence than missense mutation or small deletion.

The *KIAA0196* gene encoded strumpellin, which was an endosomal protein with 1159 amino acids highly conserved (from plants to humans) and ubiquitously expressed. It was predicted that strumpellin had multiple transmembrane domains with the N-terminal region and the central region (amino acids 241–971) comprising spectrin-repeat domains and the C-terminal region,^[7]^ and it was vital in intracellular trafficking, mitochondrial metabolism, and myelinization. Except p.I226T and p.R1035C, all the SPG8 mutations reported were in the central region. It was predicted that the spectrin-repeat domain may be a mutation hotspot and an important functional domain of strumpellin. The *KIAA0196* gene c.1128delG (p.L376fs) mutation of the proband was just in the central region of the strumpellin, which made it pathogenic. The p.L376fs mutant strumpellin might be pathogenic through a dominant-negative effect or loss-of-function-mediated haploinsufficiency. However, further studies need to be conducted to illustrate the mechanism how c.1128delG (p.L376fs) mutation causes disease.

## 4 Conclusions

This study identified a novel KIAA0196 c.1128delG (p.L376fs) mutation in a Chinese patient with SPG8. This was perhaps the first frameshift mutation of *KIAA0196* gene, resulting in the earliest onset of SPG8 in the world. Gene sequencing is a powerful diagnostic tool to identify a causal mutation in genetically heterogeneous HSP.

## 5 Method

This study was performed in accordance with the Declaration of Helsinki regarding the ethical principles for medical research involving human subjects.

## Acknowledgments

We thank the patient and his family for their kind cooperation.

## Author contributions

**Conceptualization:** Yingying Shi, Jiewen Zhang.

**Methodology:** Weiwei Qin.

**Supervision:** Shujian Li.

**Visualization:** Zhongcan Chen.

**Writing – review and editing:** Limin Ma.
